# Supplementary material for: Digital data-based strategies: A novel form of better understanding COVID-19 pandemic and international scientific collaboration
Source: PLoS One. 2021 Apr 1;16(4):e0249280. doi: 10.1371/journal.pone.0249280 (PMC8016224; doi:10.1371/journal.pone.0249280)
Supplement: Attachment — Submitted filename: Response to Reviewers.docx [file pone.0249280.s001.docx]

**Journal Requirements:**

*Please review your reference list to ensure that it is complete and correct. If you have cited papers that have been retracted, please include the rationale for doing so in the manuscript text, or remove these references and replace them with relevant current references. Any changes to the reference list should be mentioned in the rebuttal letter that accompanies your revised manuscript. If you need to cite a retracted article, indicate the article’s retracted status in the References list and also include a citation and full reference for the retraction notice.*

*When submitting your revision, we need you to address these additional requirements.*

Dear Editor, thanks first for all valuable suggestions. we have double-checked all reference:

(1) the original reference, #5 and #6 were removed because we cannot find them through PubMed and WoS:

#5.	Leydesdorff L, Wagner CS. International collaboration in science and the formation of a core group. Journal of Informetrics. 2008;2(4):317-25. doi: 10.1016/j.joi.2008.07.003.

#6.	Wagner CS, Leydesdorff L. Network structure, self-organization, and the growth of international collaboration in science. Research Policy. 2005;34(10):1608-18. doi: 10.1016/j.respol.2005.08.002.

We replace the new reference #5 and #6 as follows:

5.	Kinsella CM, Santos PD, Postigo-Hidalgo I, Folgueiras-Gonzalez A, Passchier TC, Szillat KP, et al. Preparedness needs research: How fundamental science and international collaboration accelerated the response to COVID-19. PLoS Pathog. 2020;16(10):e1008902. Epub 2020/10/10. doi: 10.1371/journal.ppat.1008902.

6.	Bell BP, Damon IK, Jernigan DB, Kenyon TA, Nichol ST, O'Connor JP, et al. Overview, Control Strategies, and Lessons Learned in the CDC Response to the 2014-2016 Ebola Epidemic. MMWR Suppl. 2016;65(3):4-11. Epub 2016/07/09. doi: 10.15585/mmwr.su6503a2.

(2) the original reference, #9 and #11 were removed because we cannot find them through PubMed and WoS:

9.	Waltman L, van Eck NJ, Noyons ECM. A unified approach to mapping and clustering of bibliometric networks. Journal of Informetrics. 2010;4(4):629-35. doi: 10.1016/j.joi.2010.07.002.

11.	Abbasi A, Hossain L, Leydesdorff L. Betweenness centrality as a driver of preferential attachment in the evolution of research collaboration networks. Journal of Informetrics. 2012;6(3):403-12. doi: 10.1016/j.joi.2012.01.002.

We replace the new reference #12 and #14 as follows:

12.	Khan A, Choudhury N, Uddin S, Hossain L, Baur LA. Longitudinal trends in global obesity research and collaboration: a review using bibliometric metadata. Obes Rev. 2016;17(4):377-85. Epub 2016/02/13. doi: 10.1111/obr.12372.

14.	Sgro A, Al-Busaidi IS, Wells CI, Vervoort D, Venturini S, Farina V, et al. Global Surgery: A 30-Year Bibliometric Analysis (1987-2017). World J Surg. 2019;43(11):2689-98. Epub 2019/08/07. doi: 10.1007/s00268-019-05112-w.

Because the Reviewer #1 gave us a suggestion to add references in Introduction part for lines 21-29, so the original reference #9 and #11 were changed to the #12 and #14.

*1. Please ensure that your manuscript meets PLOS ONE's style requirements, including those for file naming. The PLOS ONE style templates can be found at:*

*https://journals.plos.org/plosone/s/file?id=wjVg/PLOSOne_formatting_sample_main_body.pdf and*

*https://journals.plos.org/plosone/s/file?id=ba62/PLOSOne_formatting_sample_title_authors_affiliations.pdf*

According to the style requirements of *PLOS ONE*, we double-checked and revised the corresponding content, including the "Title" page, font type and size, Figure Citations, Figure Captions, Tables and Table Citations, Reference Citations, Acknowledgments, and References.

*2. We suggest you thoroughly copyedit your manuscript for* *language usage, spelling, and grammar. If you do not know anyone who can help you do this, you may wish to consider employing a professional scientific editing service.*

First, thanks Editor giving us this suggestion to help us to improve our manuscript for language usage and readability. We carefully checked and revised our paper sentence by sentence. Thank you again.

Dr. Henan Zhao edited our manuscript. The new file "Revised Manuscript with Track Changes" shows the changes by using track changes. And a clean copy of the edited manuscript will be uploaded with the new submission.

*3. We note that Figure 1 in your submission contains map images which may be copyrighted.*

Although most of the figures were obtained through programming with R software, we are very grateful for this suggestion. Finally, we accepted the suggestion b and chose the map form the USGS National Map website to replace the copyrighted map figures, and recreated the Fig 1. Furthermore, we have also added a description of referencing maps from USGS National Map website in the "Data Analysis & Visualization" of the "Methods" section to express our gratitude to the website.

*4. Please include your tables as part of your main manuscript and* *remove the individual files. Please note that supplementary tables should be uploaded as separate "supporting information" files.*

Thanks first. We add the Table 1 into the manuscript as a part of it and remove the individual file simultaneously. In addition, we do not have supplementary files, so we have no separate "supporting information" file to upload this time.

**Reviewers' comments:**

**Reviewer #1:**

*1. Add references in Introduction part for lines 21-29.*

Dear professor, thank you for your work to help us improve our manuscript. Because in the first submission, we forgot to add the line number into our paper, in the revised version, we add new references to the two paragraphs with lines between 21-29 to improve the credibility and readability of our paper.

We add the new reference #8 and #9 to illustrate that the factors affecting the pandemic are diverse and very complex.

8. Wilder-Smith A, Freedman DO. Isolation, quarantine, social distancing and community containment: pivotal role for old-style public health measures in the novel coronavirus (2019-nCoV) outbreak. J Travel Med. 2020;27(2). Epub 2020/02/14. doi: 10.1093/jtm/taaa020.

9. Bowers KW. Balancing individual and communal needs: plague and public health in early modern Seville. Bull Hist Med. 2007;81(2):335-58. Epub 2007/09/12. doi: 10.1353/bhm.2007.0020.

We add the reference #10 (its original reference number was #16) to illustrate that information and truths about the world are increasingly being extracted in the forms of digital signals and signs, rather than being generated from statistical processes.

*2. Statistical analysis should be explained in more detail.*

We first add more detail of statistical analysis into the "Statistical analysis" of the "Methods" section, including the statistical objects and statistical analysis packages involved in the programming software. Next, in Figs 3 and 4 in the "Results" section, we also added more detail into the statistical analysis, including the statistical methods, *p*-value and t-value. In the correlation analysis of the matrix, the programming package and code of correlation coefficient and *p*-value were introduced at the same time.

*3. In the reference section, all references should be in similar format.*

We have organized the format of the references according to your suggestions and PLOS ONE's style requirements. Thank you.

*4. English should be improved for this manuscript.*

We have carefully checked and revised our paper sentence by sentence, including language usage, spelling, and grammar. Thank you again.
